# Keratin Durability Has Implications for the Fossil Record: Results from a 10 Year Feather Degradation Experiment

**DOI:** 10.1371/journal.pone.0157699

**Published:** 2016-07-06

**Authors:** Alison E. Moyer, Wenxia Zheng, Mary H. Schweitzer

**Affiliations:** 1 Department of Biological Science, North Carolina State University, Raleigh NC 27695, United States of America; 2 North Carolina Museum of Natural Sciences, Raleigh NC 27601, United States of America; New York Institute of Technology College of Osteopathic Medicine, UNITED STATES

## Abstract

Keratinous ‘soft tissue’ structures (i.e. epidermally derived and originally non-biomineralized), include feathers, skin, claws, beaks, and hair. Despite their relatively common occurrence in the fossil record (second only to bone and teeth), few studies have addressed natural degradation processes that must occur in all organic material, including those keratinous structures that are incorporated into the rock record as fossils. Because feathers have high preservation potential and strong phylogenetic signal, in the current study we examine feathers subjected to different burial environments for a duration of ~10 years, using transmission electron microscopy (TEM) and *in situ* immunofluorescence (IF). We use morphology and persistence of specific immunoreactivity as indicators of preservation at the molecular and microstructural levels. We show that feather keratin is durable, demonstrates structural and microstructural integrity, and retains epitopes suitable for specific antibody recognition in even the harshest conditions. These data support the hypothesis that keratin antibody reactivity can be used to identify the nature and composition of epidermal structures in the rock record, and to address evolutionary questions by distinguishing between alpha- (widely distributed) and beta- (limited to sauropsids) keratin.

## Introduction

Beta-keratins are structural proteins expressed in the epidermis and epidermally derived structures of extant ‘reptiles’ and birds (sauropsids) [1–3]. Beta-keratin proteins are comprised of a high percentage of cysteine, a sulfur-containing amino acid. Cysteine readily forms disulfide bonds, which confer rigidity [4,5] and provide enhanced resistance to degradation. Beta-keratins also incorporate multiple hydrophobic residues in their primary structure which exclude water [6], one of the primary effectors of early degradation of proteins [7].

Beta-keratin makes up 80–90% of a mature feather [8,9]. Some researchers suggest feather beta-keratin is a corneous beta protein [4,10,11], but because the majority of researchers still refer to this family of proteins as beta-keratins we employ the more common term in this manuscript.

The inherent preservation potential of tissues and structures comprised of beta-keratin is relatively high, as suggested by previous studies [12–15] and indicated by the vertebrate fossil record in which the third most abundant fossils (after bones and teeth) are keratin-derived materials such as skin and feathers [16]. However, taphonomic studies are needed to understand the conditions leading to the arrest of degradation (and thus preservation) of these original ‘soft tissues’.

Here we report results from a 10 year experiment examining the effect of different burial conditions on feather preservation. To limit the number of variables tested, isolated feathers from a single Hungarian partridge were buried in sands taken from the Judith River formation and kept in the following conditions: 1) placed in 60°C incubator, watered twice weekly for 6 months, then intermittently for a total of 3 years, then allowed to dry and kept undisturbed for the remaining 7 years (60°C); 2) covered in foil and placed in a 350°C dry oven used for sterilization of microbiology glassware (350°C); 3) the whole bird with remaining feathers was buried in a drainage channel near a mountain pond. Five feathers were placed covered, in a clean aluminum dish and kept covered at room temperature for 10 years as a control. We employed transmission electron microscopy (TEM) to analyze ultrastructural integrity. To test for preservation of keratin epitopes of the feathers, i*n situ* immunofluorescence (IF) was performed using a custom-made affinity purified polyclonal rabbit primary antiserum raised against feather proteins.

To test the relative durability of pigmented vs non-pigmented keratin we compared and contrasted both white and pigmented portions of feathers from the control and 60°C conditions. Original color was impossible to determine in the 350°C remains. Finally, to extend these findings to the fossil record, we reexamined a fiber from a ~75 million year old fossil specimen *Shuvuuia deserti*, previously studied in 1999, with the custom-made primary antiserum used in this study [12].

## Results

**Fig 1:**
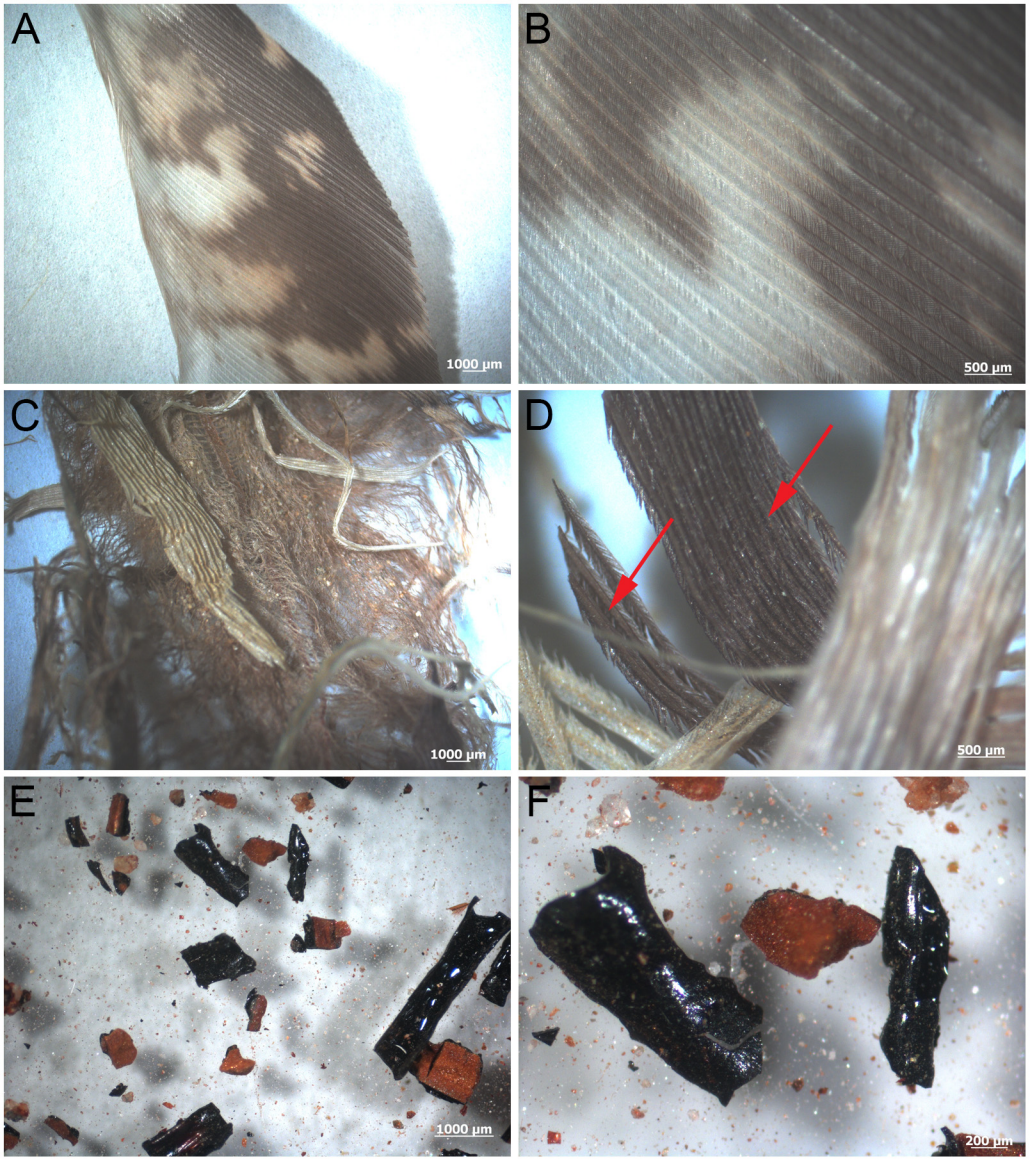
Feathers after 10 year exposure to several different environmental conditions. (A-B) Room temperature control feathers. Pigmented (reddish-brown) and non-pigmented (white to beige) patterning is visible with no signs of degradation. (C-D) Wet burial (60°C) feathers show signs of degradation and color change. Bands of pigmentation are still visible (arrows), although the white-beige parts appear more yellow and the patterning observed in control feathers are obscured. (E-F) 350°C feather pieces appear as small shiny black fragments which are associated with reddish-brown sediment. The pieces are not able to be identified to specific parts of the feather.

**Fig 2:**
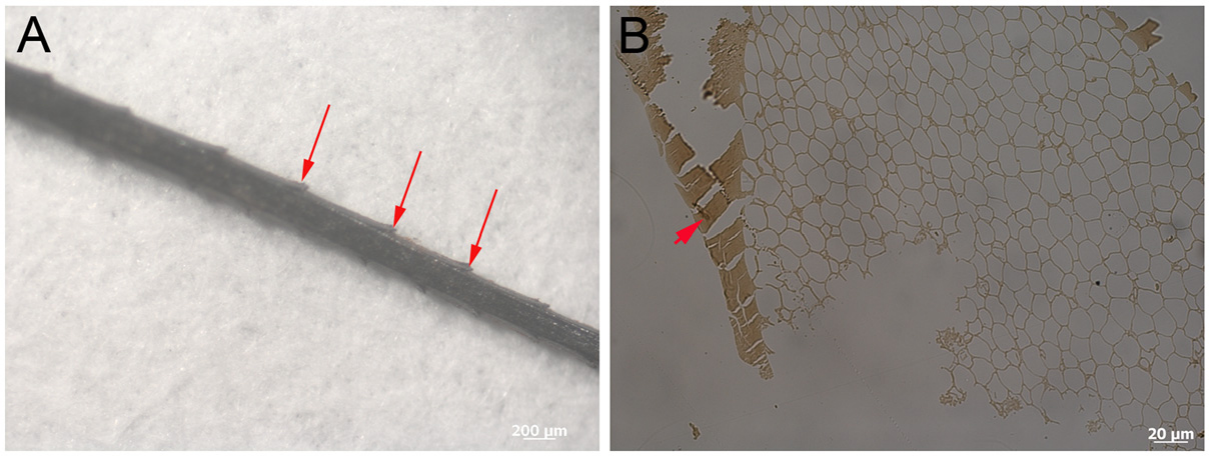
Barb fragment from 350°C feather. (A) Barb ramus retaining barbule protrusions (arrows) allows definitive identification as a barb fragment. Only the most proximal parts of the barbules can be observed where they branch from the ramus. (B) Transmitted light image of a 200nm thin section of a barb from the 350°C treated feather (similar to A) shows presence of external cortex (arrowhead) and inner pith observed as honey-comb texture.

After 10 years, optical microscopy and gross observation of Hungarian partridge (*Perdix perdix*) feathers showed considerable variation of feather structure between environments. No damage, degradation, or alteration of color or morphology was observed in control feathers kept covered at room temperature (Fig 1A and 1B). Although the wet burial (60°C) feathers (Fig 1C and 1D) retained color banding (Fig 1D arrows), the non-pigmented portions changed from white-beige to yellow, and the patterning observed in the control feathers (Fig 1A and 1B) appeared obscured in the 60°C feathers. Feathers showed obvious degradation and fraying (Fig 1C). Dry burial (350°C) feathers showed fragmentation and little original structure (Fig 1E and 1F). These feathers were very fragile and fractured during collection and sampling. Feather remnants were fragmentary and had altered from beige-white and brown to black, shiny, hollow fragments that persisted in three dimensions. Barb remains were identified in only a few cases (Fig 2A). The external cortex and internal pith of the barb can be observed in section (200nm) under transmitted light microscopy (Fig 2B). No original color could be detected in any of the 350°C feathers; feather pieces were similar to ‘carbonized remains’ observed in the rock record ([17] and the references therein), except preserved in three dimensions.

**Fig 3:**
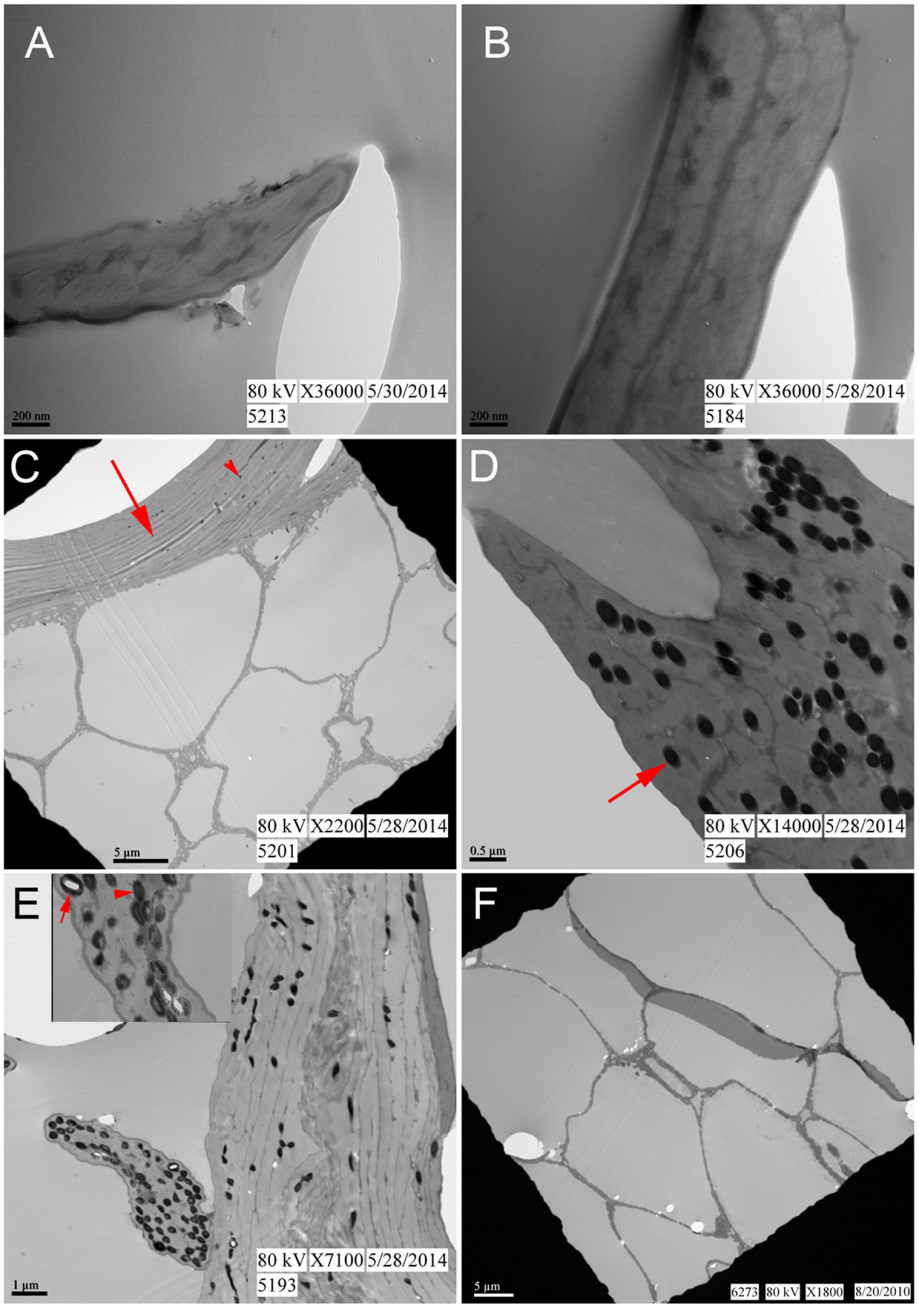
TEM images of feathers from varied conditions. (A-B) Represent unpigmented barbs from the control feather and 60°C feather respectively. (C- D) Brown barb and barbule from control feather. (C) Represents barb where cortex (arrow) and inner pith are visible. Note melanosomes (arrowhead) are sparse but present only in barb cortex. (D) Barbule with melanosomes (arrow) from control feather. (E) 60°C brown barb with barbule extending from the left side. Melanosomes are concentrated in the barbule and appear partially degraded as indicated by the less dense (arrowhead) and even ‘hollow’ (arrow) centers observed in the inset. (F) Pith from a rachis taken from the 60°C condition, embedded and sectioned separately (see Methods). Note: Whether pith derives from the rachis (F) or barb (C) is impossible to determine at this level of magnification.

Ultrastructural features of the feathers were examined using TEM. No discernible differences in ultrastructure were observed between non-pigmented (Fig 3A) and pigmented (Fig 3C and 3D) regions of the control feather, except for the presence of melanosomes in the pigmented barb (Fig 3C) and barbule (Fig 3D). There were also no discernible differences in observed ultrastructure between the white barb of the control feather (Fig 3A) and the white barb from 60°C (Fig 3B). Electron-dense microbodies consistent with melanosomes were observed in the pigmented regions of both barbs and barbules in the control (Fig 3C and 3D) and 60°C feathers (Fig 3E). The external cortex (Fig 3C arrow) and internal pith, with its characteristic honey-combed texture (Fig 3C) were observed in the pigmented barb of the control feather. The 60°C pigmented feather (Fig 3E) showed structural damage, as revealed by the scalloped texture of the feather margin. For both control and 60°C conditions, pigmented barbules demonstrated more melanosomes than cortices of the barb, as noted in the literature [18]. The melanosomes of the control feather were homogenously dense and electron opaque (Fig 3D arrow). In contrast, the microbodies in the barbule of the 60°C pigmented feather are less electron-dense, show apparent density differences relative to the control (Fig 3E arrowhead), and some are ‘hollow’ (Fig 3E arrow), suggesting partial degradation of these organelles.

**Fig 4:**
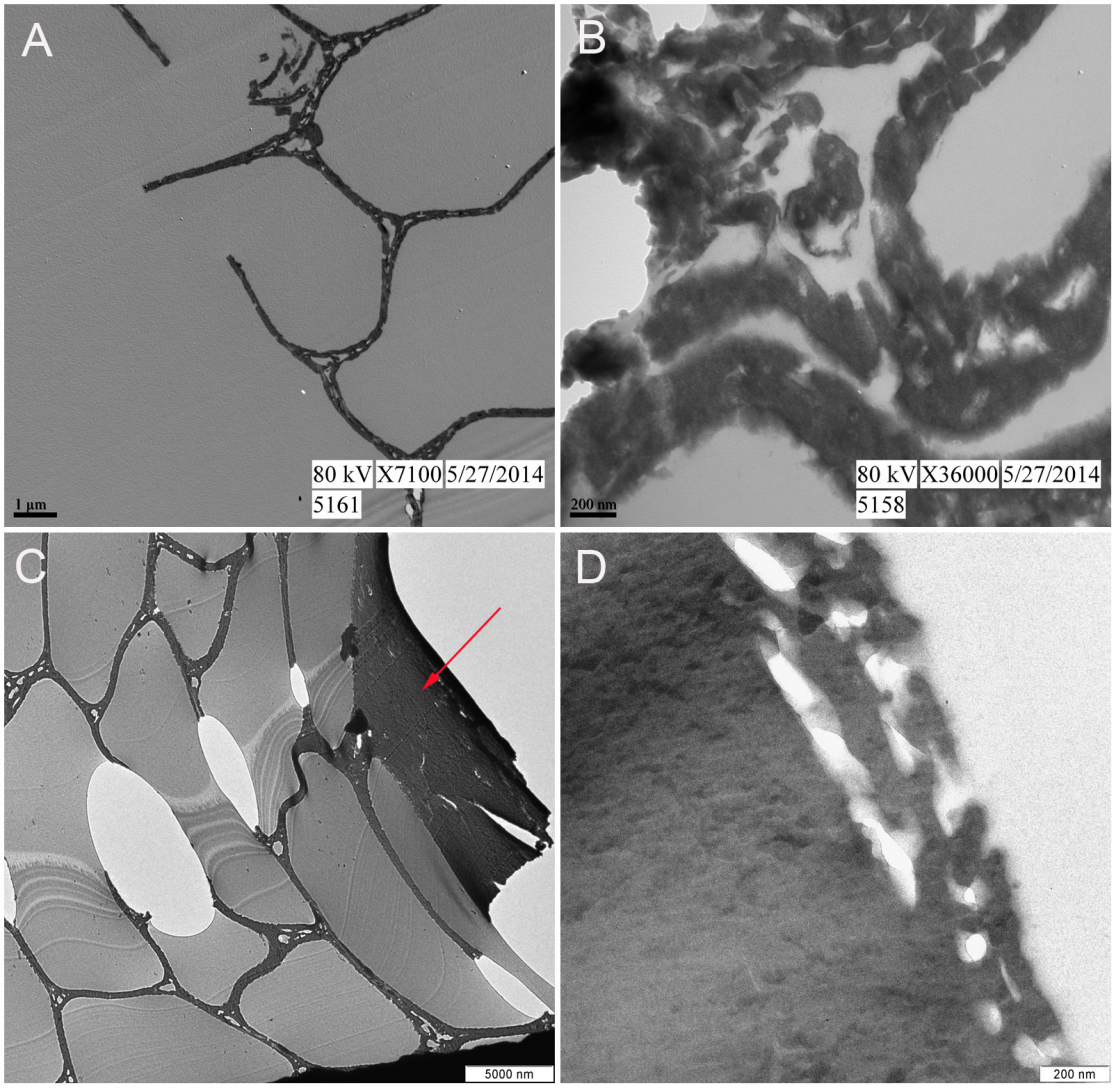
TEM images of 350°C feather fragments. (A-B) Unidentifiable piece of 350°C feather at lower and higher magnification, respectively. The honey-comb structure observed in (A) indicates it is the pith of either a barb or rachis. (C-D) Feather fragment positively identified as barb. (C) External cortex (arrow) and internal pith are observed. (D) At higher magnification no electron dense microbodies consistent with melanosomes are observed in the cortex.

Because the pith of the rachis (Fig 3F) is indistinguishable from the pith of a barb (Fig 3C) at the magnification and small sample area required by TEM, the absence of observed melanosomes in the 350°C feather (Fig 4A and 4B) may be attributed to: 1) degradation, 2) inadvertently sampling pith of barb or rachis which may not necessarily possess them, or 3) sampling regions of the feather that were originally lacking pigment. We sampled pith, but whether it derived from rachis or barb was not possible to determine. Therefore, a second feather fragment positively identified as a barb (Fig 2) was examined as well. Approximately eight fragments from the 350°C condition could be confidently identified as barbs. However, most recovered feather fragments resembled the shiny, black, nondescript pieces observed in (Fig 1E and 1F). Fig 4 shows external cortex at both lower (Fig 4C, arrow) and higher magnification (Fig 4D). Although microstructure (the outer cortex and honeycomb-like pith of the barb) is well preserved, no melanosomes are observed (see below for discussion).

**Fig 5:**
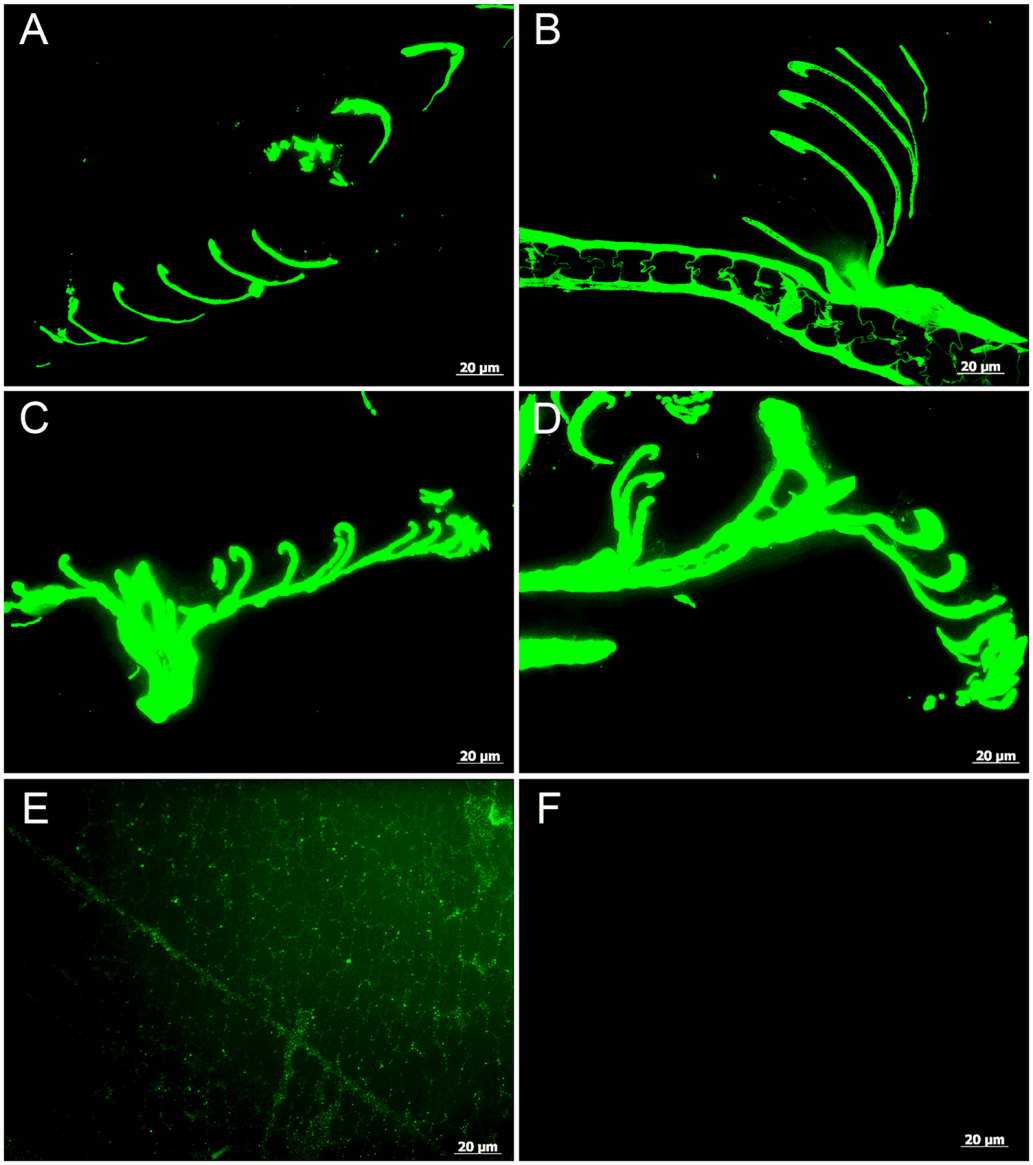
Localization of antibody antigen (ab—ag) complexes *in situ* on feathers exposed to varied conditions. (A-B) Control, (C-D) 60°C, and (E-F) 350°C feathers. White barbs (A and C) are compared with brown barbs (B and D) for both the control and 60°C conditions. There are no noticeable differences in strength of binding between the pigmented and non-pigmented barbs in either condition. Antibodies bind with greater avidity in the feathers treated at 60°C, consistent with what we have observed in samples from other experiments that are partially degraded. (E) Background signal in the 350°C condition is weak and diffuse but binding is greater than the secondary antiserum only negative control (F) and localized to the keratinous ‘struts’ within the pith.

To determine if the exceptional microstructural preservation extended to the molecular level, we employed i*n situ* immunofluorescence (IF) to test the durability of keratin epitopes under the various treatment conditions. Antibodies raised against extracts of purified feather keratin (see Methods) reacted with high specificity to all feathers tested, although binding avidity was variable. Antibody-antigen complexes, observed as green fluorescence color, strictly localized to the feather structures (Fig 5), and controls (no primary antibodies but all other conditions identical; S1 Fig) were consistently negative for binding. Intriguingly, epitope recognition was greater in the feathers treated in 60°C condition (Fig 5C and 5D) than in the control feather (Fig 5A and 5B).

Although a diffuse background signal can be seen in the 350°C feather pith (Fig 5E), the complete lack of binding in the control (no primary antibodies added) (Fig 5F and S1 Fig) supports specific localization of ab-ag complexes to feather tissues.

To test the hypothesis that pigmented feathers are more resistant to degradation than non-pigmented feathers, we compared white barbs (Fig 5A and 5C) to brown barbs (Fig 5B and 5D) within each condition. No differences in structure or binding avidity were observed between colored and white feather parts in both the control and 60°C experimental conditions.

**Fig 6:**
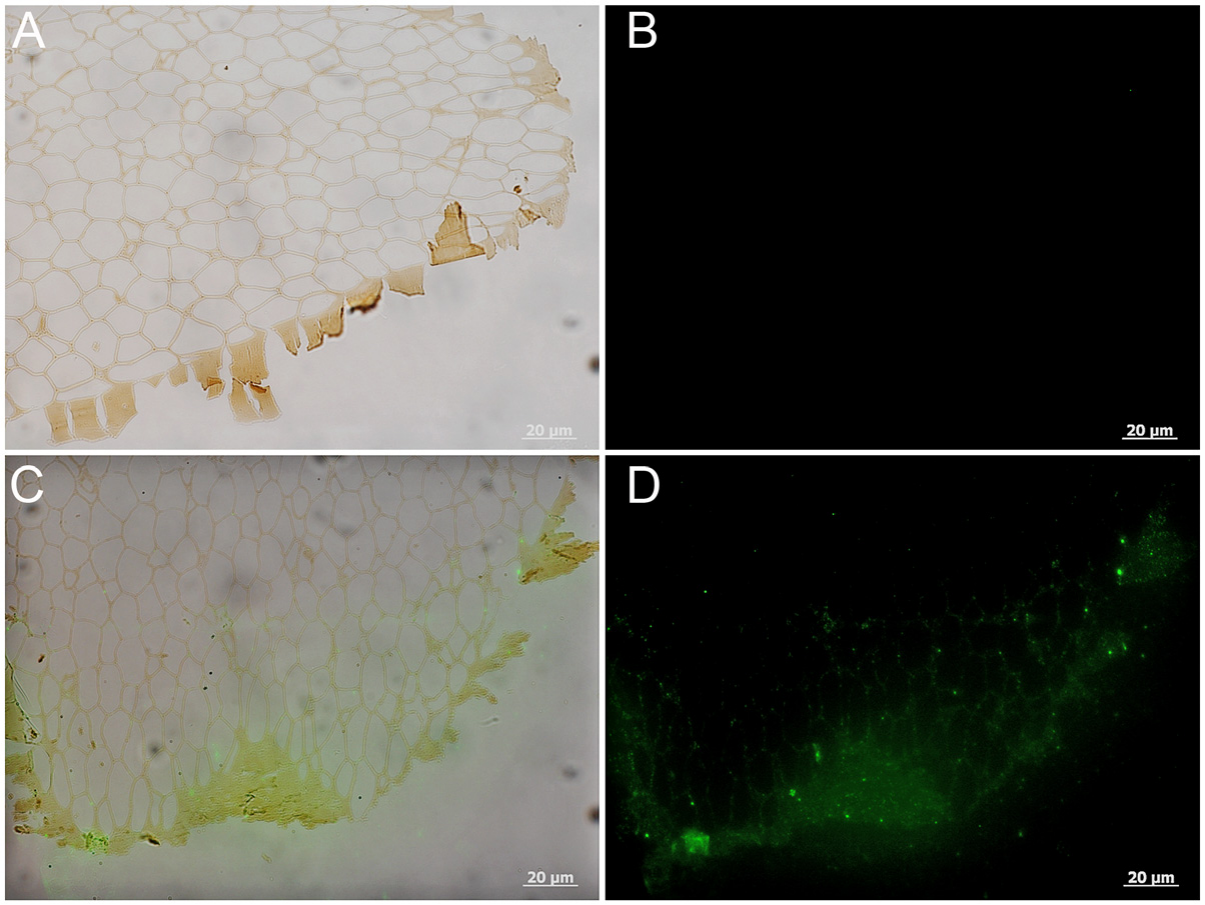
Localization of antibody antigen (ab—ag) complexes *in situ* on 350°C barb. (A-B) Secondary antibody only control shows no signal in the absence of the primary antiserum. (C-D) Positive binding of primary antibodies to the 350°C feather barb. Note that the diffuse signal is localized to the cortex (C-D) and the adjacent lattice structure of the pith.

Two pieces of feather from the 350°C condition were analyzed using IF. One piece was either barb or rachis (see above discussion) and one piece was definitively barb (see Fig 2). Although it was impossible to distinguish areas of original color in the feather treated for 10 years at 350°C, we were able to demonstrate weak positive binding of anti-feather antibodies localized to the tissues (Figs 5E, 6C and 6D). Fig 6 illustrates IF results on the feather fragment identified as a barb and shows the cortex as well as the inner pith of the barb. Antibody binding is localized to the cortex as well as the lattice structure of the pith just deep to the cortex (Fig 6C and 6D). Control conditions where no primary antibodies were added, were completely without signal (Fig 5F, S1J Fig and Fig 6B). In both samples, background signal is observed as a green ‘glow’ (Figs 5E and 6D) however binding of the primary antiserum is localized to the feather microstructure. These data support the preservation of some protein components, correlating with the exceptional microstructural integrity, but positive signal is greatly decreased compared to the other conditions.

The specificity of our custom antibodies was further demonstrated through an inhibition study. When antibodies are incubated first with excess feather protein extract to block the binding sites of the antibodies, then exposed to tissue sections, binding is reduced or eliminated (S2C and S2D Fig, for modern and S4C and S4D Fig, for fossil), testifying to the specificity of antibody-antigen interactions.

To further test specificity, we incubated the antibodies with human fingernail, and no binding was observed; however an alpha-keratin antibody applied to human fingernail reacted positively, showing the nail expresses alpha-keratin epitopes (S3A–S3C Fig). The lack of response of human nail to the custom antibody employed herein supports the specificity to tissues containing beta-keratin (S3D–S3F Fig). As in previous experiments, adding only secondary antiserum, without specific primary antibodies, controls for non-specific binding of the secondary antibody to the tissues (S3G–S3I Fig).

## Discussion

Fossil remains originally comprised of keratin have been noted since the 1800s (e.g., [19,20]), but few studies have sought to elucidate the microstructure and composition of these structures, or the mechanisms leading to their preservation. Additionally, recent discoveries and analyses of fossil feathers ([21–26] and the references therein) illustrate the need to understand mechanisms leading to the preservation of these evolutionarily significant structures. In this long term study, we examined the microstructural and molecular preservation of modern feathers subjected to varying conditions, including high heat which has been suggested as a proxy for time (e.g. [27–30]), to directly test the stability of these materials.

The small size of the barbs/barbules allowed the entirety of the structures to be observed under electron microscopy and these parts are where melanosomes are concentrated; therefore barbs were analyzed preferentially over rachises when possible. We show that after several years at 60°C in a wet burial condition, original color is transformed macroscopically (Fig 1), and feather microstructure is also altered (Fig 3). Some of the melanosomes remain embedded in the keratinous matrix, but appear partially degraded, as revealed by a loss of internal electron-opaque material within the melanosomes. We conclude that the semi-translucent nature of melanosomes in these feathers is a result of partial degradation and not the ‘hollow melanosome’ morphology characterized in the literature in some species of birds [31,32] for the following reasons: 1) No hollow melanosomes were observed in the control feathers. 2) This observation appeared in feathers *only* after exposure to wet burial conditions at 60°C for several years.

The compositional and structural differences between families of keratin proteins is significant, and as mentioned previously, mammals do not express beta-keratins, while feathers have little to no detectable alpha-keratin in mature form [33–35]. Although degraded, beta-keratin epitopes comprising the feather were capable of being detected, and in fact, antibody signal appears greater in the partially degraded feather than in the feather kept at room temperature for the same duration. We hypothesize that this increased response to antibodies is due to a taphonomic degradation pathway, similar to enzyme degradation [36], where breakage of the intramolecular bonds results in the exposure of epitopes that would otherwise be blocked.

It has been proposed that melanized feathers are more durable than unpigmented ones [37,38]; therefore we also compared microstructural and molecular degradation between brown (pigmented) and white (non-pigmented) portions of feathers, when they could be distinguished. This resistance has been attributed to the durability of melanin, the primary (or dominant) biochrome in colored feathers. Gunderson et al. (2008) concluded that non-pigmented feathers are more susceptible to degradation, but our results do not support this under the conditions we employed. No obvious differences in structural integrity were observed under TEM between white and brown regions of the feathers. They also did not differ in antibody response, showing neither increased nor decreased fluorescent signal.

Immunohistochemistry is a powerful analytical method if appropriate controls are performed in parallel. For each sample tested using *in situ* IF in this study, we confirmed specificity of our antibody response by applying the secondary antibody only and no primary antiserum. If no binding was observed, this negates the possibility of spurious (or non-specific) binding of the secondary antibody, and showed that only the binding of the primary antibody to its specific epitope, retained in the tissue, resulted in a positive signal.

In addition to this negative control, we confirmed signal specificity using additional controls. These included inhibiting the primary antiserum by exposing it to an excess of purified antigen against which the antibody was raised prior to incubation with the tissue (S2 Fig). The protein then occupied the binding sites of the antibody such that they were no longer available to bind with the test sample. Antibodies with binding sites for other proteins or epitopes would not be inhibited by beta-keratin proteins and would be free to bind fossil tissues. Therefore, signal should be reduced or completely lost in the inhibition test. Indeed, this control was negative for binding in the extant chicken feather tissue (S2C and S2D Fig).

To control for non-specific binding of the primary antibody, we applied an irrelevant antibody (S2E and S2F Fig), one to which no binding to the sample would be expected, while all other parameters were kept consistent. As we demonstrated, no signal should be observed in the presence of an irrelevant (i.e., not expected to recognize the epitopes in the test sample) antibody. Often, as in most analytical techniques, there is background signal; however signal above this background, as long as all parameters are kept consistent between sections being compared, is assigned to a positive and specific signal. In each case, the controls we employed were consistently negative for binding, supporting that all binding signal is specific to epitopes present in the samples tested.

Remarkably, even under the harshest conditions in this experiment, feather microstructure and immunoreactivity, consistent with the presence of durable epitopes of beta-keratin proteins comprising the feather matrix, were repeatedly demonstrated. Furthermore, although melanosomes were visible in both the room temperature control and 60°C wet burial feathers, melanosomes were not observed in the dry burial at 350°C remains after ten years. This may be explained if: 1) beta-keratin is more resistant to degradation than the pigment-containing organelles, 2) we sampled rachises (except for the definitive barb in Fig 2), which were unpigmented in the control feathers, thus not expected to contain melanosomes in these specific feathers, or 3) the feather fragments analyzed for this condition were originally non-pigmented; however, the starting condition for these feathers were a mix of pigmented and unpigmented regions.

Data supporting molecular mechanisms that result in the preservation of these feathers under long-term degradation experiments is beyond the scope of this paper. However, we propose hypotheses for future testing that are suggested by our initial results. Rapid mineralization, usually microbially mediated, has been invoked as a primary agent for early stabilization of organic components before degradation can occur [39,40]. In this process, microbial overgrowth of decaying organics results in the secretion of exopolymeric substances (EPS) that are negatively charged [41,42], facilitating the deposition of positively charged mineral ions. However, this mechanism does not directly apply to this experiment, particularly to the feather kept at 350°C, because it is doubtful that microbes would grow in such an environment. An alternative hypothesis is that these colored feathers underwent ‘melanin leaching’ during degradation [15]. Although we have no we have no morphological evidence for pigment containing melanosomes in any 350°C feather examined after this 10 year experiment, we propose that melanin pigment may have leached out of the organelles to disperse throughout the feather, acting as a fixative agent to prevent further degradation (J. Lindgren, pers. comm). This hypothesis is amenable to future testing.

In the recent literature, microbodies associated with fossil feathers have enthusiastically been ascribed to melanosomes. These structures are depicted in SEM (scanning electron microscopy) images as confluent, over-lapping structures ([25] and references therein). Contrary to those studies, the melanosomes observed in these partridge feathers, as well as chicken feathers from a previous study [43], were distinct and non-overlapping (Fig 3D) even after partial degradation (Fig 3E) and did not display the density and arrangement depicted in SEM images of structures purported to be melanosomes in fossil feathers. These contrasting observations illustrate the need for more taphonomic studies that explore the degradation of feathers and their microstructural components.

**Fig 7:**
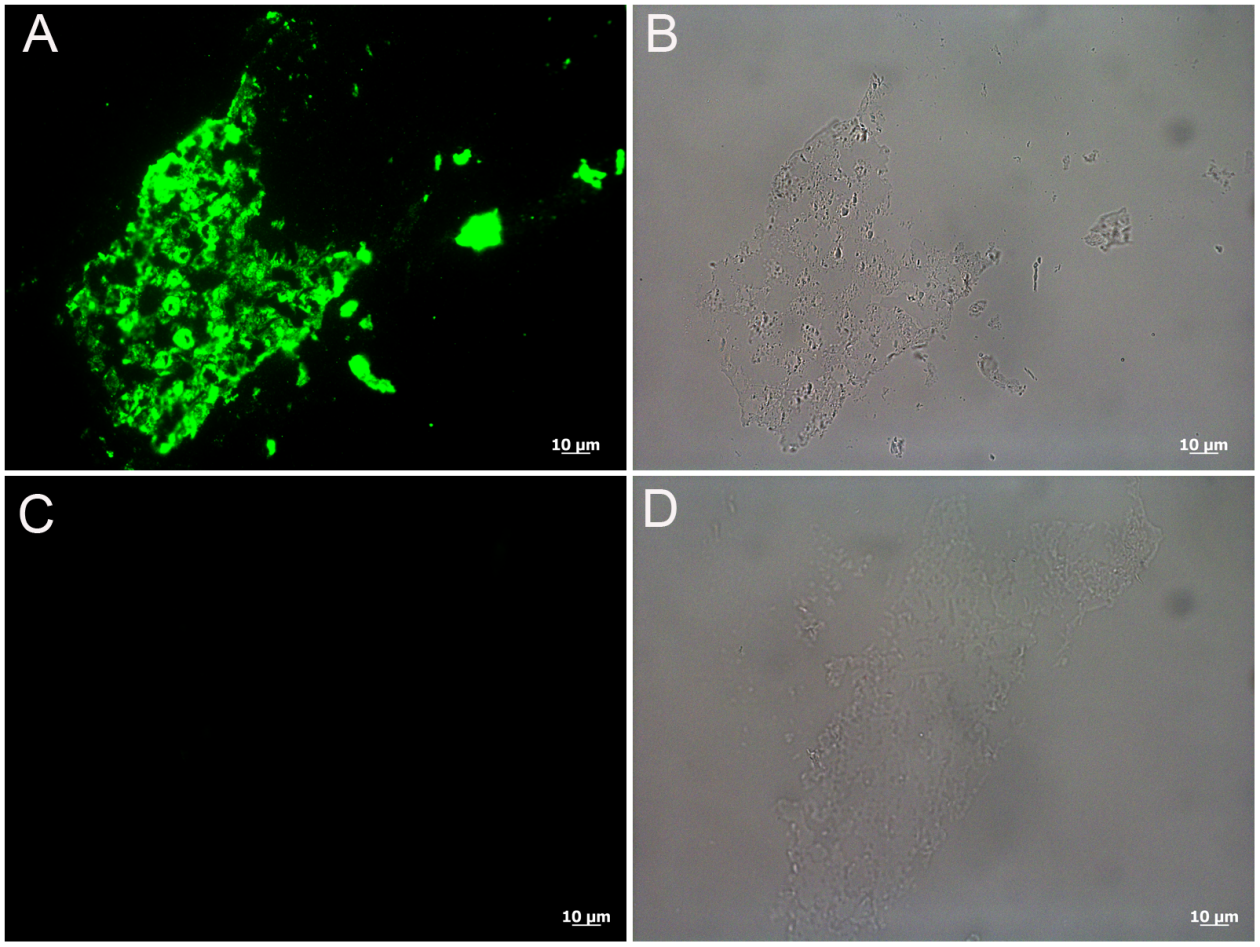
Localization of antibody antigen (ab—ag) complexes *in situ* on *Shuvuuia deserti* (IGM 100/977) filament. (A) Shows positive binding of the anti-chicken feather antiserum to the fossil tissue as indicated by the green fluorescent signal. (B) Demonstrates that the binding in A) is localized specifically to the tissue. (C and D) Secondary antiserum only control is negative and shows that the positive signal in A) is not due to spurious antibody binding.

Because the data herein support the durability of keratin-derived structures as well as the persistence of original keratin epitopes under the harshest condition, the application of antibodies to identify and/or differentiate structures in the rock record is supported. Therefore we applied our antibodies to a specimen already shown to demonstrate microstructural and molecular preservation. In 1999, data were published to support the hypothesis that the fiber collected from the cervical region of the articulated *Shuvuuia deserti* specimen, an alvarezsaurid from Mongolia, was a feather-like structure [44]. These data included microscopic and immunohistochemical consistencies between the fossil and modern feather material [12]. Positive binding, localized to the *S*. *deserti* sample, was also observed using the custom-made primary antiserum employed in this study (Fig 7A and 7B). The control condition, where no primary antibody was exposed to *S*. *deserti* fibers, but all other conditions kept identical to the test specimens, showed no signal (Fig 7C and 7D and S4E and S4F Fig). Furthermore, the inhibition control (discussed above), in which the primary antibody was applied after being exposed to extracted feather protein, resulted in a greatly reduced signal in the fossil tissue (S4C and S4D Fig) compared to the positive control (S4A and S4B Fig). This also testifies to the specificity of the signal to epitopes consistent with beta-keratin. TEM images of the hollow, fossil fiber (refer to Fig 4 in [12]) demonstrated filaments 3nm in diameter, consistent in size with modern beta-keratin filaments [3]. No electron-dense microbodies consistent with melanosomes were observed, and the fibers were universally white *in situ*.

The positive binding observed in the fossil sample further demonstrates the specificity of our antibody as well as its potential to be used for paleontological studies. Repeating the results from the original study with a second and custom-made beta-keratin-specific antibody validates the previous results and attests to the endogeneity of the feather-like epitopes in an ancient sample. It also demonstrates the stability of a sample in LR white embedding medium at ambient conditions for many years. Finally no structures consistent with melanosomes were observed in this ancient specimen (see SEM and TEM results Figs 3 and 4 respectively in [12]), similar to the results from the 350°C condition in this study, but the preserved samples demonstrated microstructural and molecular integrity consistent with modern feathers.

## Conclusions

Using heat as a proxy for time [27–30] we show that molecular and microstructural features of feathers comprised of beta-keratin proteins persist for long time periods under conditions harsher than most fossil material will endure; our data also support their persistence in the fossil record over exceedingly long time spans at milder conditions. Thus, beta-keratin proteins are exceptional target molecules for paleontological studies.

As previously mentioned, numerous studies of fossil feathers have recently interpreted small microstructures as color-imparting melanosomes, based in part on the resistance to degradation of the pigment melanin, but in modern feathers, melanosomes are embedded in a keratin matrix, which our data suggest should also preserve, and under our experimental conditions preserve better than melanosomes. If keratin protein is more resistant to degradation than melanosomes, which has not been identified in the previously published studies of ancient feather samples, alternative hypotheses of a microbial origin for these preserved microstructures remain. Because of the wide distribution of microbes [45], their association with all degrading organic matter (e.g. [40,46]), and their demonstrated longevity (e.g. [47]), microbes indeed may be a more parsimonious explanation for their source. Thus, a microbial hypothesis must be eliminated with chemical data before interpretations of ancient color can be made.

We show that immunohistochemistry is one technique that can detect keratin and may be used in future studies to differentiate between a keratinous and a microbial origin for these materials. Furthermore the durability of keratin in feathers may be extended to other keratinous structures in the rock record that may be studied by these techniques. Both epidermally derived keratinous structures and expressions of color are key innovations in evolutionary history, and certainly contribute to the success of ancient organisms. Characterizing taphonomic transitions will provide more accurate interpretations of ancient organisms and the world with which they interacted. Only by studying and observing these processes in the lab can we then make predictions and assumptions about the fossilization processes that took place millions of years ago.

## Methods and Materials

The Hungarian partridge used in this study was taken in a hunt from private land. The bird was donated to the study and the landowner is since deceased. The species is neither protected nor endangered and is quite plentiful on the Montana prairies. No permissions were required because the bird was taken by a private citizen and was not killed for this study.

Feathers from a single Hungarian partridge (*Perdix perdix*) with rust, grey and white wing feathers, and body feathers of grey to rust (Fig 1) were subjected to four environmental conditions spanning a 10 year observation period. Feathers were plucked from a fresh-killed bird, covered to exclude dust, and maintained unaltered at room temperature (control feathers). For the other three conditions, feathers from the same bird were buried in fine-grained Judith River (JR) sand collected from a dinosaur excavation site to maintain as much consistency as possible. The burial conditions were; 1) buried in saturated sand in a container with holes for draining, intermittently watered with distilled water (once daily to weekly on average), and incubated at 60°C for approximately 3 years, then allowed to dry and kept buried at RT for the remaining 7 years (wet burial), 2) buried in saturated sand, then baked at 350°C uninterrupted for 10 years (dry burial) and 3) the whole bird with attached feathers was placed in an ~1.5m hole in which ~6–8 inches (~15–20 cm) of JR sands were placed, then the bird carcass, then covered with remaining JR sands. The hole was dug in a drainage channel surrounded by rich peaty soil adjacent to a mountain pond (stream burial).

Temperature has been suggested in the literature to be a proxy for time in the degradation of organic molecules with the general trend that warmer and wetter conditions are detrimental to molecule survival [27–30,48,49]. The temperatures employed in this study were chosen based on availability to instruments at a facility where temperature could be maintained for an extended period of time.

Upon completion of the experiment, all feathers were kept covered at room temperature until analysis. Although disarticulated bones from the fourth condition were recovered, no evidence of feathers remained. Barbs and barbules, rather than rachises, were sampled when possible for comparison between pigmented and unpigmented portions of the feathers.

### Transmission electron microscopy (TEM)

Samples of feathers from the first three conditions of this taphonomic experiment, as well as a reddish-brown chicken feather and human fingernail (see Specificity Controls below), were collected and fixed in 10% neutral buffered formalin for a minimum of one hour at room temperature, followed by a PBS (phosphate-buffered saline) wash. From the control and 60°C wet burial conditions, white feather barbs and brown barbs were prepped and analyzed separately to assess any differences in degradation between pigmented and non-pigmented feathers. Samples were dehydrated in two changes of 70% ethanol, then incubated in (2:1) LR white: 70% ethanol to equilibrate, and infiltrated by incubating in three changes of LR white embedding medium. Each specimen and embedding medium were placed in a gelatin capsule, capped to exclude oxygen, and allowed to polymerize for 48 hours at 60°C. This embedding polymer was chosen because it is permeable, allowing penetration of antibodies (see below).

A piece of rachis from the 60°C wet burial condition was embedded in Spurr’s medium to demonstrate that the internal pith structure of a rachis and barb are indistinguishable. The sample was fixed in a solution of formaldehyde and glutaraldehyde (4F:1G) overnight, rinsed with 0.1M PBS (pH 7.2), then post fixed with 1% osmium tetroxide in 0.1M PBS for 24 hours. After washing in PBS, the sample was serially dehydrated in acetone (30%, 50%, 70%, 90%, 100%) for 15 minutes at each concentration, then equilibrated in a graded series of Spurr’s:acetone solutions (1:3 for 6 hours, 1:2 overnight, 1:1 for 3 hours and 3:1 for 3 hours). Following an overnight infiltration of the sample with pure Spurr’s, the sample was embedded in a silicone mold and cured at 70°C for 48 hours.

Embedded feathers from each condition were sectioned to 90nm using a Leica EMUC6 ultra-microtome with a diamond knife, then mounted on 200 mesh copper grids and stained with 15% methanolic uranyl acetate and Reynold’s lead citrate prior to imaging. Microstructure was capable of being visualized without staining in the 350°C feather (Fig 4C and 4D). The feathers were imaged with a Zeiss Leo 912 TEM 100KV accelerating voltage with a Proscan 2048X2048 CCD (Olympus Soft Imaging System) (Fig 4C and 4D) or an Erlangshen ES1000W Model 785 TEM coupled to a CCD 11Megapixel High-speed Digital Camera (Gatan Microscopy Suite (GMS) software) (Figs 3, 4A and 4B).

### *In situ* immunohistochemistry (IHC)—Immunofluorescence (IF)

Using the same LR white-embedded specimens sectioned for TEM (described above), 200nm sections were taken for IHC. Sections were transferred to a 6-well Teflon coated slide, dried on a plate warmer (45°C) and then allowed to fully dry overnight at 42°C.

The primary antiserum for this study is an affinity-purified, polyclonal rabbit anti-chicken feather antibody produced by Bio-Synthesis, Inc (Lot Number: AB1312-1). Except where specifically mentioned, all antibody tests reported here employ this custom-made antiserum. The immunogen was protein recovered from a white chicken primary flight feather, extracted with a buffer comprised of 20mM Tris-HCl (pH = 8.5), 2.6 M thiourea, 5 M urea and 5% 2-mercaptoethanol (BME) [50]. One milliliter of the extracted protein solution was dialyzed (using 3500 MW snakeskin dialysis tubing) then resuspended in 15 mL PBS buffer (pH = 6.8). Two laboratory rabbits (BSYN 6733 & 6734) at Bio-Synthesis, Inc. were injected subcutaneously and intramuscularly, then boosted subcutaneously multiple times over 12 weeks. The final 12 week serum collection was purified using a protein-bounded affinity column to concentrate the antibody and remove non-specific components of the serum. Mature feathers are unique among keratinous structures in that approximately 80–90% of the protein is beta-keratin [8,9], therefore it can confidently be assumed the extracted protein used to immunize is primarily, if not entirely beta-keratin. Antibody reactivity (observed as green fluorescent signal) marks the presence of epitopes of feather beta-keratin expressed in tissues which are recognized by the antibody.

*In situ* IF was conducted to localize binding of the beta-keratin antibodies to epitopes within the feather samples. All incubations were performed at room temperature unless noted otherwise and separated by 2x5 minute washes using IHC phosphate buffered saline (PBS). To expose epitopes, sections were incubated for 15 min with 25 μg/μL Proteinase K in 1X PBS at 37°C, followed by 3x10 minutes with 0.5 M ethylenediaminetetraacetic acid (EDTA) pH 8.0, which participates in antigen retrieval. Sections were incubated for 2x10 min with 1mg/mL NaBH_4_, also an antigen retrieval method as well as to reduce autofluorescence, then with 4% normal goat serum (NGS) in IHC PBS for four hours to block nonspecific binding sites. The primary antiserum was diluted in dilution buffer (1% bovine serum albumin (BSA), 0.1% cold fish skin gelatin, 0.5% Triton X-100, 0.05% sodium azide, 0.01M PBS (pH = 7.2–7.4)) at 1:100 dilution (unless otherwise noted), applied to all test samples and incubated overnight at 4°C. Controls consisting of identical tissues sectioned and treated as above were incubated with dilution buffer containing no primary antibodies in tandem with and under identical conditions as the test samples. This controlled for non-specific binding of secondary antibodies or detection agents.

After incubation with primary antiserum (or dilution buffer only), all remaining incubations were separated by two washes in PBS with 5% Tween20 wash buffer for 10 minutes, followed by two 10-minute washes in PBS to remove unbound antibody. All sections, including controls, were then incubated with secondary antibody (biotinylated goat anti-rabbit IgG(H+L) from Vector Laboratories, BA-1000, Lot-Y1228) diluted 1:500 in secondary dilution buffer (0.01M PBS (pH = 7.2), 0.05% Tween 20) for 2 hours. Fluorescein Avidin D (Vector laboratories, A-2001, Lot-W1124), diluted 1:1000 in secondary dilution buffer and incubated in darkness, was used to detect antibody-antigen complexes. Slides were mounted with Vectashield mounting media (Vector laboratories, H-1000, Lot-Y0417), coverslips applied, and sections visualized using a Zeiss Axioskop 2 plus biological microscope.

Images were captured using an AxioCam MRc 5 (Zeiss) with 10x ocular magnification on the Axioskop 2 plus using Axiovision software package (version 4.7.0.0). For each antibody test, all images were acquired with the same data acquisition parameters unless otherwise noted here. Control and 60°C feather images were collected at 30ms exposure and the 350°C images were captured at 100ms exposure. At 100ms, the control and 60°C images were over-exposed and therefore the exposure was reduced. All other data acquisition parameters were kept identical amongst the respective images. The images in Fig 6 of the 350°C barb were captured at the same magnification and exposure as the images of the other 350°C sample in Fig 5, however required minimally modified acquisition parameters because of slightly higher background signal.

### Antibody specificity controls

In addition to the application of all buffers and solutions as above, but without primary antiserum applied (secondary antiserum only control), additional steps to control for non-specific binding and to demonstrate specificity of antigen retrieval were performed. This included the application of the primary antiserum as described above, to which was added extracted chicken feather proteins (1:200 dilution in a protein solution with ~1.75 mg of protein determined using a Pierce^®^ BCA protein assay kit (Thermo Scientific, Product# 23227, Lot# OI193596)) to block binding sites and inhibit incubating antibodies. Then, this primary antiserum (now blocked by the protein solution) was incubated with brown chicken feather. A second control for specificity included application of an irrelevant primary antibody (anti-human elastin (courtesy of R. Mecham), 1:500) not expected to have epitopes cross-reacting with feather antibodies.

To further demonstrate the specificity and application potential of our custom-made antibody, we tested it against fossil material from *Shuvuuia deserti* (IGM 100/977) [44] previously studied for the presence of feather-like structure and composition [12]. The same samples, already embedded and trimmed for sectioning, from the original study were used. In addition to maintaining consistency, this allowed for us to test the stability of an embedded sample remaining at ambient conditions for ~16 years. Tissues were prepped and immunohistochemical analyses performed identical to modern tissue (see Methods above) but in a separate lab with equipment and materials designated for working with ancient samples only. Thin sections (200nm) of *S*. *deserti* were tested against the anti-chicken feather antibody and a secondary antiserum only control was run in parallel. Images were captured at 80ms exposure and acquisition parameters were set to -.27 for brightness, 2.72 for contrast, 1.81 for gamma. Images were captured at the same magnification but altered acquisition parameters than modern sections because of different background signal. The antibody inhibition control, as described above for modern tissue, was also performed on a second sample of fossil tissue. These images were captures at the same exposure and magnification as the other fossil sample. However, the primary antiserum was applied at 1:200 dilution, to keep consistent with the inhibition dilution, and acquisition parameters were set to -.09 for brightness, 9.00 for contrast, 1.45 for gamma.

To show that our antiserum does not cross-react with alpha-keratin epitopes (thus refuting the hypothesis that *in situ* binding results from human contamination), human fingernail was embedded and sectioned as described above and used as antigen in identical experiments. Fingernail sections were exposed to our custom-made antiserum (1:200 dilution) as well as a polyclonal rabbit anti-alpha-keratin antibody (1:200 dilution) (provided by L. Knapp).

To demonstrate repeatability and accuracy of results, we performed all assays an average of three times.

## Supporting Information

S1 Fig Immunological staining results for the secondary antibody only (negative) controls of feathers exposed to varied conditions. (A-D) Room temperature control feathers. (E-H) 60°C wet burial feathers and (I-J) 350°C dry burial. White barbs (A and B, E and F) and brown barbs (C and D, G and H) are both shown. The brightfield filter (A, C, E, G) reveals the presence of the tissue and the FITC filter (B, D, F, H, J) demonstrates the absence of fluorescence indicating a negative result. Because all other conditions were identical (for the same tissues tested in Fig 5), this controls for non-specific binding of the secondary antibody to the tissues. No signal observed in this control supports that the binding observed in Fig 5 is specific to the tissue recognized by the anti-feather primary antiserum.
(JPG)

S2 Fig Immunological staining results from the specificity controls of the primary antibody on extant tissue. (A and B) The positive control using brown chicken feather tested against the anti-chicken feather primary antiserum (1:200 dilution). (C and D) Brown chicken feather subjected to the primary anti-chicken feather antibody after inhibition by incubating with extracted chicken feather protein (1:200 dilution, See Methods). The absence of binding, observed as the lack of fluorescence in D), shows that the antigen retrieval is specific to the keratinous tissue, because all binding sites were occupied by the specific antigen prior to exposure. (E and F) Feather incubated with anti-human elastin. As expected, binding is negative and negates the possibility that positive binding of the anti-chicken feather antibody is spurious or non-specific. (G and H) Negative control in which only the secondary antibody was applied, no primary antiserum, showing no binding as expected. Note: the artefact observed in the top right corner of image C is an air bubble.
(TIF)

S3 Fig Immunological staining results from human fingernail tissue. (A-C) Sectioned human fingernail tissue tested against anti-rabbit alpha-keratin as a positive control. (E-F) Fingernail tissue tested against the anti-rabbit chicken feather protein. (G-I) Negative control in which ‘secondary only’ antibody applied. This control demonstrates that the custom made anti-chicken feather protein antibody does not bind human alpha-keratin thus ruling out a positive response due to human contamination.
(JPG)

S4 Fig Immunological staining results from the inhibition control of the primary antibody on *S*. *deserti* tissue. (A and B) Positive control of fossil tissue tested against the primary anti-chicken feather antiserum (1:200 dilution). (C and D) Inhibition control where fossil tissue was incubated with the primary antiserum after it had been exposed to pure chicken feather antibodies (1:200 dilution) to block the active binding sites. Binding is greatly reduced demonstrating the positive binding observed in A and B is specific to epitopes consistent with beta-keratin proteins. (E and F) Negative control in which no primary antiserum, secondary antiserum only, was applied to the tissue.
(TIF)
