# Supplementary figures and images for: Keratin Durability Has Implications for the Fossil Record: Results from a 10 Year Feather Degradation Experiment
Source: PLoS One. 2016 Jul 6;11(7):e0157699. doi: 10.1371/journal.pone.0157699 (PMC4934732; doi:10.1371/journal.pone.0157699)

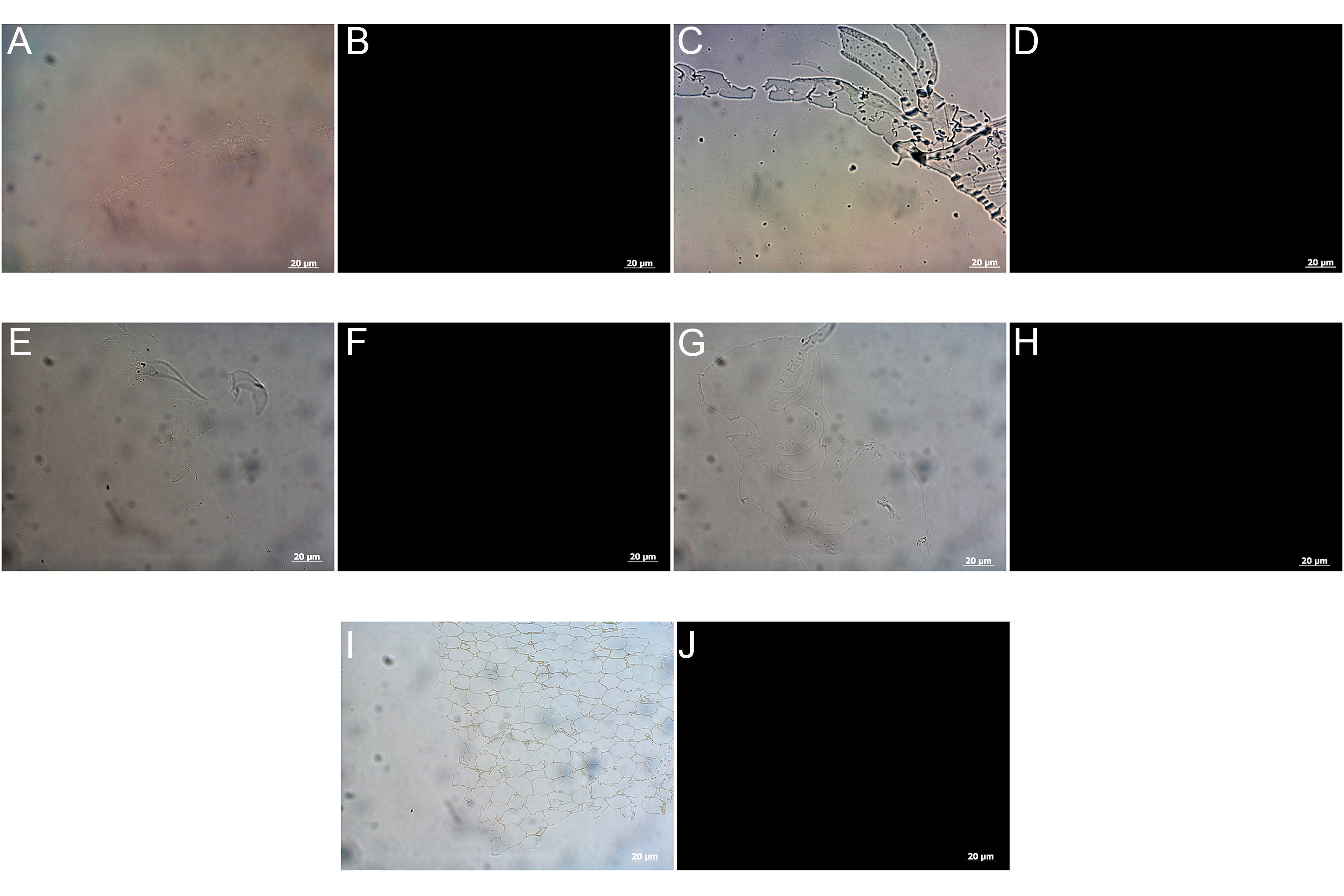

Supplement: S1 Fig — (A-D) Room temperature control feathers. (E-H) 60°C wet burial feathers and (I-J) 350°C dry burial. White barbs (A and B, E and F) and brown barbs (C and D, G and H) are both shown. The brightfield filter (A, C, E, G) reveals the presence of the tissue and the FITC filter (B, D, F, H, J) demonstrates the absence of fluorescence indicating a negative result. Because all other conditions were identical (for the same tissues tested in Fig 5), this controls for non-specific binding of the secondary antibody to the tissues. No signal observed in this control supports that the binding observed in Fig 5 is specific to the tissue recognized by the anti-feather primary antiserum. (JPG) [file pone.0157699.s001.jpg]

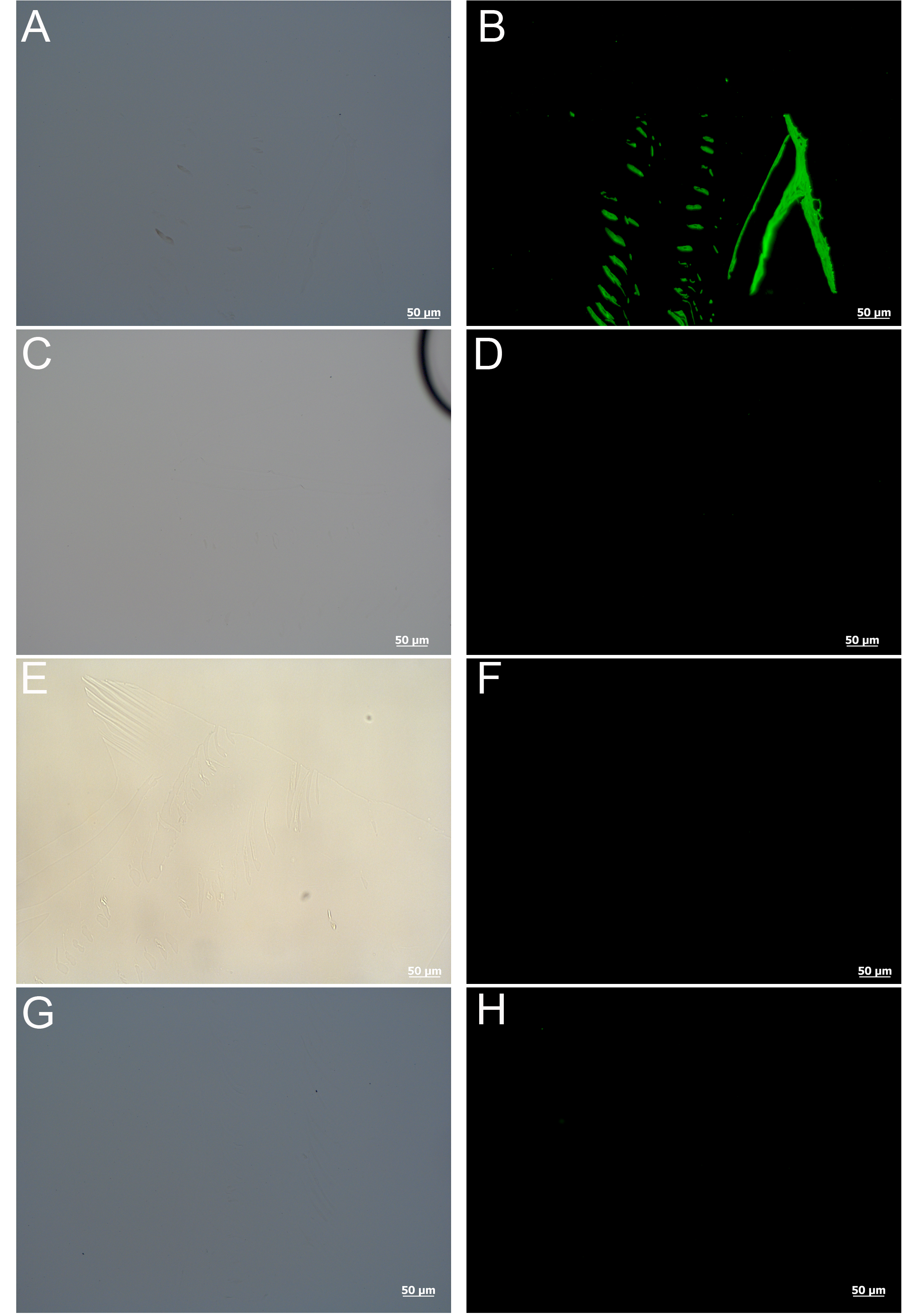

Supplement: S2 Fig — (A and B) The positive control using brown chicken feather tested against the anti-chicken feather primary antiserum (1:200 dilution). (C and D) Brown chicken feather subjected to the primary anti-chicken feather antibody after inhibition by incubating with extracted chicken feather protein (1:200 dilution, See Methods). The absence of binding, observed as the lack of fluorescence in D), shows that the antigen retrieval is specific to the keratinous tissue, because all binding sites were occupied by the specific antigen prior to exposure. (E and F) Feather incubated with anti-human elastin. As expected, binding is negative and negates the possibility that positive binding of the anti-chicken feather antibody is spurious or non-specific. (G and H) Negative control in which only the secondary antibody was applied, no primary antiserum, showing no binding as expected. Note: the artefact observed in the top right corner of image C is an air bubble. (TIF) [file pone.0157699.s002.tif]

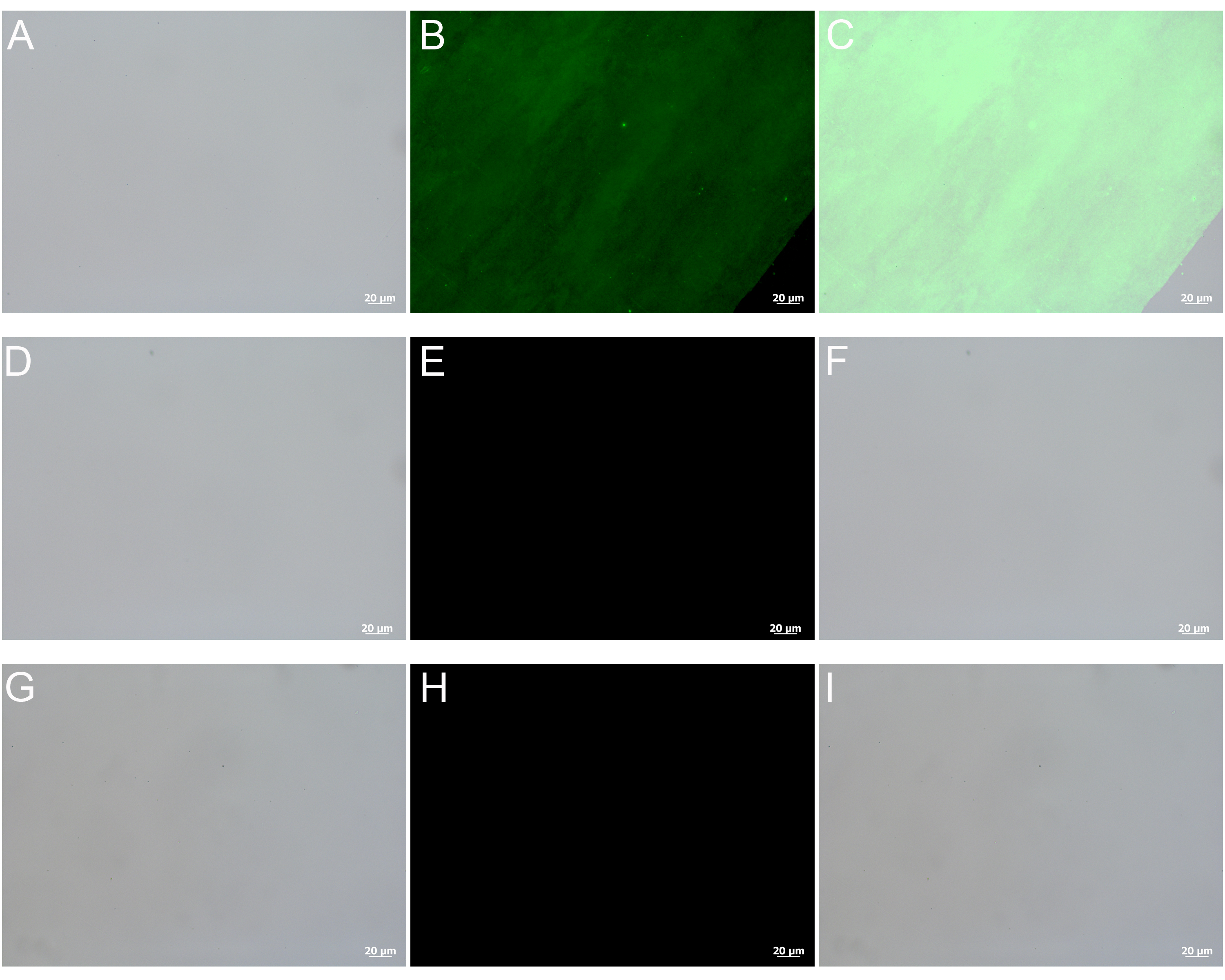

Supplement: S3 Fig — (A-C) Sectioned human fingernail tissue tested against anti-rabbit alpha-keratin as a positive control. (E-F) Fingernail tissue tested against the anti-rabbit chicken feather protein. (G-I) Negative control in which ‘secondary only’ antibody applied. This control demonstrates that the custom made anti-chicken feather protein antibody does not bind human alpha-keratin thus ruling out a positive response due to human contamination. (JPG) [file pone.0157699.s003.jpg]

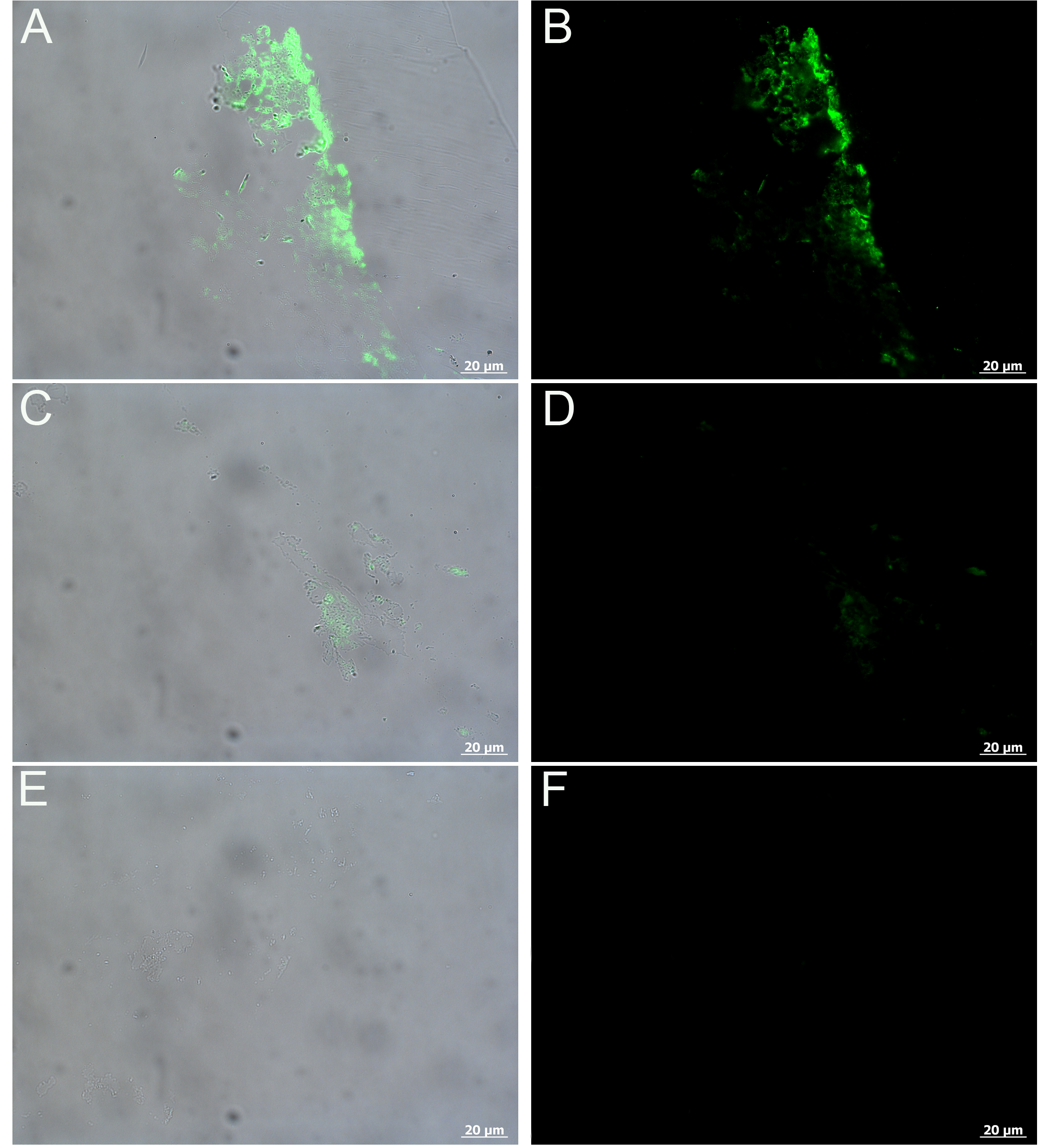

Supplement: S4 Fig — (A and B) Positive control of fossil tissue tested against the primary anti-chicken feather antiserum (1:200 dilution). (C and D) Inhibition control where fossil tissue was incubated with the primary antiserum after it had been exposed to pure chicken feather antibodies (1:200 dilution) to block the active binding sites. Binding is greatly reduced demonstrating the positive binding observed in A and B is specific to epitopes consistent with beta-keratin proteins. (E and F) Negative control in which no primary antiserum, secondary antiserum only, was applied to the tissue. (TIF) [file pone.0157699.s004.tif]
